# The commitment of French midwifery teachers to doctoral studies: A qualitative study based on experience feedback

**DOI:** 10.18332/ejm/189422

**Published:** 2024-06-27

**Authors:** Céline Mahieu

**Affiliations:** 1 Department of Midwifery Studies, Rouen University Hospital, Rouen, France

**Keywords:** midwifery teacher, doctoral studies, commitment, midwifery research, PhD

## Abstract

**INTRODUCTION:**

In 2014, nine percent of French midwifery teachers were enrolled in doctoral studies or already had a doctoral degree, although they are only required to have a Master's degree. Doctoral studies last at least three years and require high intellectual work. This study aimed to evaluate the motivational sources underlying the commitment of French midwifery teachers to their doctoral studies, and to identify the factors involved in managing their doctoral studies, family life, and professional life.

**METHODS:**

The study has a qualitative design. In 2021, fifteen midwifery teachers enrolled in doctoral studies participated in the study and were interviewed. The data were analyzed using thematic content analysis.

**RESULTS:**

Analysis of data revealed a wide range of motivational sources of French midwifery teachers. Interviews showed that French midwifery teachers hoped to gain recognition as a medical profession by contributing to research to improve the quality of care for women and newborns. French midwifery teachers were successful in managing their doctoral studies, family life, and professional life. They showed resilience in the face of the various obstacles they encountered during their doctoral studies, in particular, the lack of funding for their studies and the lack of recognition of their doctoral work.

**CONCLUSIONS:**

The study highlights the commitment of French midwifery teachers to their doctoral studies and to developing midwifery research. However, they lack the time, resources, and funding for their doctoral work.

## INTRODUCTION

The commitment of French midwifery teachers to their doctoral studies has been little evaluated. A study conducted in 2014 listed the university degrees held by French midwifery teachers^1^. At that time, out of 241 midwifery teachers and midwifery school directors, 9% were doctoral students or held a PhD. However, currently, there is no requirement to hold a doctorate to teach midwifery in France. It is important to add that the population of French midwifery teachers mainly consists of women with an established family life who return to studying while maintaining their professional careers.

Two factors help to contextualize the reasons for French midwifery teachers choosing to pursue a doctoral degree. The first one is that the midwifery students taught by midwifery teachers are now enrolled in a training program that is being universitized and the second one is that the national council of universities (CNU), a consultative and decision-making body, has opened a new section for midwifery.

The Bologna agreements of 1999 initiated the universitization^2^ of vocational training: *‘Universitization can be said to occur when institutions that dispense knowledge in a professional sector, the knowledge itself and the teachers who dispense it are so to speak absorbed by the university … The training staff themselves are confronted with a new dominant status, that of research teacher, which requires them to have a PhD to qualify.’*

Before the move to universitization in France, midwifery training was conducted in hospital schools. Since then, midwifery training bodies have gradually been integrated into universities. Midwifery training lasts five years, and the diploma obtained on completion of the course is recognized as a Master’s degree. The law of 25 January 2023, aimed at developing the training of midwives^3^, provides for a third cycle of studies leading to the state diploma of midwifery doctor after the thesis defence, as for other medical professions:

*‘In France, midwifery represents the specialized field of midwives. They practice as health professionals, having the right to consult and prescribe medications and have autonomy of practice, as outlined in the Public Health Code. Midwives’ traditional areas of practice are obstetrics and neonatal pediatrics. Since 2009, they have also been practicing gynecology and, from 2016, are qualified to provide treatment and care for abortion.’*

Midwifery students are supervised by midwifery teachers who, from 2016, were required to hold a Master’s degree if they were unable to obtain a management diploma before the closure of the only midwifery management school in 2011.

In France, the CNU manages the recruitment and career-monitoring of research teachers. The CNU comprises 92 sections, each representing an academic discipline. A specific midwifery section was created in 2019, recognizing this discipline at a scientific level. Since then, the first 40 midwives with PhDs have qualified in this section and are eligible for a research teaching position in midwifery. Seven of these 40 midwives are currently lecturers, and three are university professors^3^. For the time being, there is no midwifery research team. The first midwives with PhDs obtained doctorates in other disciplines. Sauvegrain et al.^3^ proposed this definition:

*‘Midwifery research encompasses all research activities related to midwifery (the discipline practiced by midwives): empirical, clinical, and fundamental research in midwifery; research on midwifery education and the profession, its evolution and societal role; and finally, research on the organization of healthcare systems and services specifically involving midwives. The fields of perinatal, gynecological, and sexual health, as well as medical education, are included in midwifery research. The methodologies employed are diverse, reflecting the variety of scientific disciplines of the researchers.’*

Midwifery research is beginning to develop in France, as in other countries^4-7^. A doctorate represents the third cycle of higher studies. In France, three types of doctorates exist: 1) a doctorate of professional practice for medicine, pharmacy, dentistry, and soon midwifery; 2) a doctorate of business administration; and 3) a doctorate of research for scientific research.

The focus of this study is the doctorate of research. The duration is a minimum of three years, to which a maximum of three years can be added. Doctoral students prepare for a research career while being integrated into the scientific community. The doctorate is not limited to writing a thesis. In France, depending on the doctoral school, doctoral students must carry out additional activities such as participating in doctoral training, writing articles, participating in seminars, and teaching^8^. The learning approach involved in a doctorate is different from other training. It is characterized, among other things, by autonomy and the ability to organize the workload. To assist in this learning process, a doctoral contract is drawn up between the doctoral student and a thesis supervisor or two co-supervisors. Only some doctoral students receive funding. Having a regular job while studying for the doctorate increases the risk of dropping out due to problems in reconciling work and family life^9^.

The aim of this study was to evaluate the motivational sources underlying the commitment of French midwifery teachers to their doctoral studies and to identify the factors involved in managing their doctoral studies, family life, and professional life.

## METHODS

This research was carried out as part of a research thesis^10^, sought to evaluate the motivational sources of French midwifery teachers regarding their doctoral studies and to identify the factors involved in managing their doctoral studies, family life, and professional life. In particular, we asked midwifery teachers how they successfully maintained their commitment to doctoral studies despite an already-established family life and professional life. We used the terms ‘path’ and ‘trajectory’ defined by Sapin et al.^11^, namely, that a life path is made up of several life trajectories that interact with each other.

### Study design and study population

The study design was based on a qualitative method with interviews exploring the portrayals of midwifery teachers regarding their doctoral studies. Interviews seem to be suitable for capturing portrayals^12^.

### Study setting, participant recruitment period

In January 2021, the 35 midwifery schools in France were contacted by email or telephone. In March 2021, 213 midwifery teachers and 35 directors were professionally active. Among them, 16/213 midwifery teachers and 3/35 directors held a PhD, and 14/213 midwifery teachers and 4/35 directors were studying for a PhD. The directors provided the contact details of the doctoral students. The population of midwifery teachers included some school directors with teaching responsibilities. A total of 15/18 midwifery teachers eligible for this study agreed to participate in the interviews. Data saturation was reached with these 15 interviews^13^.

### Data collection and data interpretation

An interview guide was written with the following starting point: ‘You are a midwifery teacher, and I would like you to talk to me about your commitment to your doctoral studies’. The interviewees were able to ‘tell the story’ of their commitment to doctoral studies^14^. A semi-directive interview guide made it possible to ask about key points, such as their sources of commitment (motivation, goals), perseverance in their commitment (the organizational structure adopted corresponding to the cognitive commitment and the actions carried out with regard to the behavioral aspect), and their experience of the commitment (the emotional aspect).

Demographic data were collected, including the marital status and the number of children of the participants. A biographical approach was used to analyze multiple events by considering the different trajectories and contexts followed by the participants. It also allowed to analyze their experiences and the influence they had on their current actions and portrayals^15,16^. In a phenomenological approach, participants were asked to make a reflexive analysis of the acts carried out and the decisions taken^17^. Thus, the interviewer asked questions about intentional events that occurred as well as about passive memory events, even if we are aware that this is not holistic. The interviews were conducted in 2021, by videoconference or telephone. Interviews lasted one hour and twenty-five minutes on average. They were recorded, transcribed, and anonymized. In this article, verbatims and quotations from French publications have been translated by the author.

We developed an encoding grid based on the different elements obtained from a review of the literature, such as the indicators of commitment according to Pintrich et al.^18^ through the behavioral, cognitive, and emotional manifestations of commitment in training, as well as the factors influencing commitment in doctoral studies. This encoding was consistent with the interview guide. These themes were encoded using Nvivo 12 software. Then, we conducted a content analysis^19^. We sought to reduce desirability and cognitive biases by establishing a climate of trust with each participant and by ensuring the anonymity of the data collected.

### Ethics

An information form and a written consent form were drawn up according to the guide for research of the *Centre National de la Recherche Scientifique*. These two documents were validated by the Data Protection Officer (DPO) of our University. The DPO registered the study (n°2021.018) and specified that it was not necessary to make an additional declaration to obtain ethical approval. Before the interviews, the participants were informed that they could withdraw from the project at any time. All persons who agreed to be interviewed gave their informed consent.

## RESULTS

The baseline characteristics of the participants (all women) are presented in Table 1.

**Table 1:** Baseline characteristics of participants, 2023^10^

*Participant*	*Profession*	*Age group (years)*	*Number of years of doctoral studies at the time of the first interview*	*Doctoral discipline*	*Number of years between Master’s and the start of doctoral studies*
P1	Midwifery teacher	51–55	0	Human and Social Sciences	4
P2	Midwifery teacher	36–40	1	Sciences, Technologies, and Health	8
P3	Midwifery teacher	51–55	4	Human and Social Sciences	3
P4	Midwifery teacher	46–50	7	Human and Social Sciences	4
P5	Midwifery teacher	46–50	3	Human and Social Sciences	0
P6	Midwifery teacher	36–40	4	Sciences, Technologies, and Health	8
P7	Midwifery teacher	31–35	3	Sciences, Technologies, and Health	4
P8	Midwifery school director	46–50	2	Human and Social Sciences	6
P9	Midwifery teacher	51–55	2	Human and Social Sciences	Not known
P10	Midwifery school director	41–45	4	Sciences, Technologies, and Health	0
P11	Midwifery teacher	41–45	2	Sciences, Technologies, and Health	0
P12	Midwifery teacher	46–50	1	Sciences, Technologies, and Health	4
P13	Midwifery teacher	51–55	1	Sciences, Technologies, and Health	6
P14	Midwifery teacher	41–45	5	Human and Social Sciences	0
P15	Midwifery school director	36–40	3	Human and Social Sciences	12

### Motivational sources of commitment to doctoral studies

Thirteen of the fifteen participants stated that they had enrolled in a doctoral program because they were personally motivated. All stated that they had enrolled in a doctoral program due to more or less explicit recommendations. Some were strongly encouraged to do doctoral studies in order to take up certain professional assignments, especially in the current context of the gradual integration of midwifery schools into the university system:

*‘There was also the support from the institution, in inverted commas, because in our university there is a strong desire to join a health faculty … To be a fully-fledged department, there also needs to be someone with a PhD.’* (P12)

In midwifery schools that have already integrated into the university system, the pressure to hold a PhD is strong as teacher-researcher positions will be created gradually, and there will not be many of them. The participants believed that a PhD would give them the legitimacy to teach Master’s students. Some considered that in the future, a doctorate would be mandatory for midwifery teaching posts and decided to anticipate policy requirements:

*‘It makes sense as regards the future, possibly; I have a little over 20 years left to work, and it is not a requirement to be a teacher right now, but at some point, it certainly will be.’* (P11)

Ten participants said they had enrolled in a doctoral program to develop their careers. Some felt a need for professional fulfillment and development. For some, a PhD seemed to be a crucial aspect in ensuring the recognition of midwifery in terms of its medical status:

*‘As long as midwives are not academics and do no research, it will be very hard to make the same claims as medical doctors, pharmacists, and dentists.’* (P8)

*‘If we want legitimacy in universities, at some point we have to go for it … Midwives often complain about not having a place … but no one will hand it to us on a plate. If we want it, we have to take it, and to take it, we have to do something … We must not miss this opportunity. I don’t think we’re missing it, I think we’re taking it.’* (P13)

Six participants believed doctoral studies strengthened their professional skills in terms of teaching or in terms of research in midwifery or disciplines overlapping with their professional practice:

*‘Doing doctoral studies ultimately encourages you to publish and to know a bit about the mechanics of doing so. The aim of doctoral studies for me is … being able to pass on this knowledge to students afterwards.’* (P12)

### Behavioral manifestations of commitment to doctoral studies

The participants showed their commitment to doctoral studies through their behavior, their actions, and their decisions. First of all, they had to choose their doctoral discipline, their thesis subject, and their thesis supervisor. The subject of the thesis was often linked to care, sometimes related to women’s health, and sometimes to students. They reported the absence of a research team dedicated to midwifery, obliging them to enroll in another doctoral discipline that was linked to midwifery:

*‘There is no discipline focused on the perinatal period … and in the end, it’s a subject … at the crossroads of several other disciplines.’* (P15)

Different doctoral schools propose different programs, with a wide difference in the number of teaching hours, ranging from 4 to over 100 hours. Also, different disciplines have different requirements in terms of scientific publications. In the science and technology discipline, theses are ‘article theses’, often with a requirement of two published articles and a third at the submission stage during the doctoral studies:

*‘I’m doing an article thesis, so until now I’ve focused on writing articles, and that implied virtually one article every year.’* (P10)

In human and social sciences, the publication of articles is not required, but doctoral students are encouraged to focus on their thesis and to publish mainly with a view to a subsequent CNU appointment:

*‘In the discipline I’ve chosen, what is important is the thesis manuscript and the report, so they don’t necessarily ask for an article.’* (P14)

Commitment was also demonstrated by other behavioral indicators such as managing efforts and difficulties, seeking assistance and networking within the academic world. Some participants sought help and, for example, obtained partial funding for their thesis. Only four out of 15 were allocated time for their doctoral studies. Two received grants to enable them to attend a conference. Some sought help and motivational support. For some, this meant planning meetings with their thesis supervisor; for others, it meant meeting other doctoral students for extracurricular activities.

### Cognitive demonstrations of commitment to doctoral studies

When they enrolled in doctoral studies, some of the participants had already researched the various activities involved in these studies, while others had negotiated funding for their studies with their employers or other organizations. Depending on these and other parameters, they had envisaged a certain length of time for their studies. However, some of them had to rethink their organization and the timeframe envisaged for the doctoral studies after discovering unexpected activities:

*‘When I registered … I didn’t understand the work it represented at all.’* (P8)

Almost all of the participants expected their doctoral studies to last longer than the minimum of three years. The majority lived with a partner and had children. Nine had children at home. Three had health problems. All said that they lacked time. The time allowed for doctoral studies was often affected by other life trajectories, notably personal (health problems, young children) and professional aspects. They said that their priority was their family and children. However, they were forced to work on their thesis to the detriment of their personal life, particularly in terms of sleep:

*‘I start working once everyone is in bed.’* (P15)

**Figure 1:**
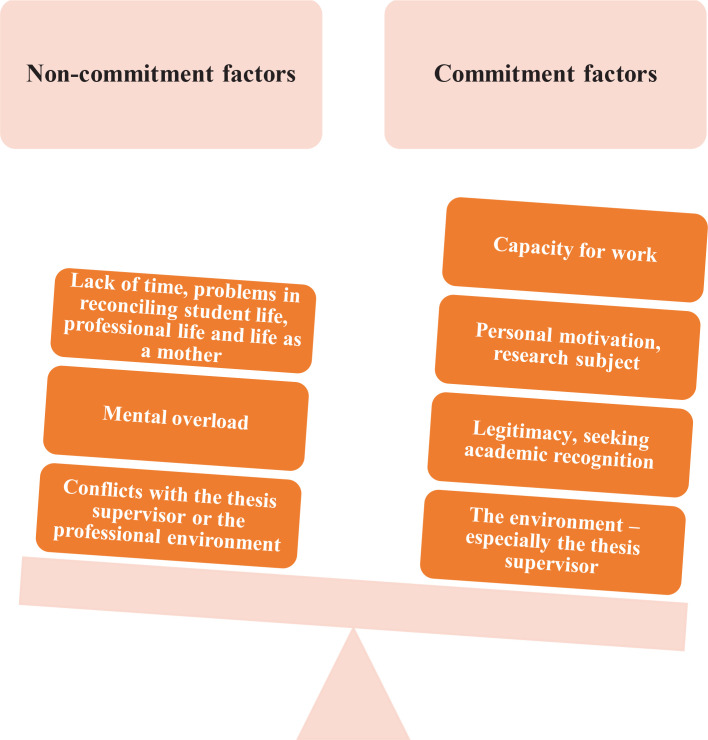
Factors influencing the commitment and non-commitment of French midwifery teachers to their doctoral studies, 2023^10^

As regards their doctoral studies fitting in with their midwifery teaching, they all declared that they did not want the time spent on their studies to negatively impact their support for midwifery students. They carried out a reflexive analysis of the factors influencing their commitment or non-commitment to their doctoral studies. Figure 1 gives an overview of the keywords used by the participants to describe factors of commitment and non-commitment to doctoral studies.

Through the words used by the participants, an emotional and affective aspect emerged to describe their commitment to their doctoral studies.

### Emotional demonstrations of commitment to doctoral studies

The participants considered doctoral studies and their commitment to their doctoral studies extremely important. The words used to describe the value of doctoral studies were largely positive. Doctoral studies also evoked feelings of doubt and anxiety about the difficulties encountered during these years. A lack of time and problems reconciling their doctoral studies with their professional life were often the cause of these difficulties. Some experienced mental overload and exhaustion:

*‘I never said I was going to give up the thesis, but there were times when I felt so overwhelmed that he could see I was on the verge of burn-out all the same.’* (P8)

All participants retained an interest in their thesis subject, mainly due to the encouragement some received from their families, the support others got from their thesis supervisors, and the encouragement others received from the doctoral students they met.

## DISCUSSION

The main finding of this study is the strong commitment of midwifery teachers to doctoral studies since the universitization of midwifery teaching in France. This is in line with the definition of Bourdoncle^2^. Midwifery teachers were perhaps anticipating a policy requirement for doctoral degrees, aimed at aligning the status of midwifery teachers with that of other teachers in higher education. The study participants were keen to pursue their research work after their doctoral studies with the objective of improving women’s health. The commitment of midwifery teachers to their doctoral studies reflects their sense of responsibility regarding their professional development, in particular by choosing to enroll in doctoral programs^20^. They found meaning in their commitment to doctoral studies, and this encouraged them to persevere with their doctoral studies^21^. They hoped that the development of midwifery research would encourage recognition of their medical status. The state of midwifery research in France was evaluated in a recent study^22^.

This observational study shows a high level of commitment of midwifery teachers to their doctoral studies, evidenced by behavioral, cognitive, and emotional indicators^18^. Behavioral indicators include choosing a doctoral program, perseverance with doctoral studies, the way efforts are managed, and knowing how to ask for help when needed. Cognitive indicators include setting goals to be achieved steadily in order to progress and self-assessment by adopting a reflexive attitude on the progress of their learning work. Emotional indicators include a sense of pride and enthusiasm regarding their doctoral studies. The midwifery teachers interviewed dedicated a great deal of their personal time to the various doctoral activities. Few obtained funding for their studies. All indicated a work overload because they had to reconcile their doctoral activities with their professional work, to which they were also highly committed, and their family life, which they wished to preserve above all. The tension between doctoral studies and personal life is one of the main reasons for dropping out of doctoral studies^23^. Doctoral students who have to work at the same time lack the time to write their thesis and communicate the results of their research at conferences or in scientific journals^24^, all the more so when the doctoral students are women^25^. This was also the case for midwives carrying out research in France^22^. The midwifery teachers interviewed received little funding to attend conferences or to publish in journals requesting article processing charges. As reported by Demeester and Chantry^26^ and also Sauvegrain et al.^3^:

*‘Until recently, the French healthcare system did not provide midwives with the opportunity to have specific time allocated for research*.*’*

Doctoral students had to be self-regulating if they wanted to make progress during their doctoral trajectory, as doctoral studies are a form of autonomous learning. Some feared a mental overload. Some of the factors that have a positive influence on doctoral commitment have been identified. These factors include support and encouragement especially from the thesis supervisor and their close circle^27,28^, self-regulation skills^29^, self-efficacy^30^, personal motivation^31^, as well as the important value it has for the profession. Support from other doctoral students and thesis supervisors is a protective factor against stress and burnout during doctoral studies^32,33^.

### Strengths and limitations

The main strength of this qualitative study is that it allowed us to gather the portrayals of participants based on their own experiences during their doctoral studies^34^. Jodelet^35^ described portrayals as mental realities, interpretations or versions of reality, with different meanings depending on the individual or the social group to which the individual belongs. In this study, commitment was assessed in interviews and not by observing actions; however, the aim was to collect portrayals of commitment to doctoral studies, and the methodological approach of interviews made this possible.

## CONCLUSIONS

The universitization of midwifery teaching in France is an important motivational source underlying the commitment of midwifery teachers to their doctoral studies. However, it is also important to acknowledge the difficulties midwifery teachers encounter during their doctoral studies. These difficulties could be offset by strengthening the available resources, in particular the funding of doctoral studies, allowing midwifery doctoral students to focus their time and attention on the many aspects of their midwifery doctoral studies.

## Data Availability

The transcripts of the interviews, although anonymized, are not publicly available in order to protect the participants.
